# Oil palm natural diversity and the potential for yield improvement

**DOI:** 10.3389/fpls.2015.00190

**Published:** 2015-03-27

**Authors:** Edson Barcelos, Sara de Almeida Rios, Raimundo N. V. Cunha, Ricardo Lopes, Sérgio Y. Motoike, Elena Babiychuk, Aleksandra Skirycz, Sergei Kushnir

**Affiliations:** ^1^Embrapa Amazonia Ocidental, Empresa Brasileira de Pesquisa Agropecuária, Manaus, Brazil; ^2^Department of Phytotechnology, Federal University of Viçosa, Viçosa, Brazil; ^3^Department of Sustainable Development, Vale Institute of Technology, Belém, Brazil

**Keywords:** oil palm, *E. guineensis*, *E. oleifera*, germplasm, hybrid, breeding

## Abstract

African oil palm has the highest productivity amongst cultivated oleaginous crops. Species can constitute a single crop capable to fulfill the growing global demand for vegetable oils, which is estimated to reach 240 million tons by 2050. Two types of vegetable oil are extracted from the palm fruit on commercial scale. The crude palm oil and kernel palm oil have different fatty acid profiles, which increases versatility of the crop in industrial applications. Plantations of the current varieties have economic life-span around 25–30 years and produce fruits around the year. Thus, predictable annual palm oil supply enables marketing plans and adjustments in line with the economic forecasts. Oil palm cultivation is one of the most profitable land uses in the humid tropics. Oil palm fruits are the richest plant source of pro-vitamin A and vitamin E. Hence, crop both alleviates poverty, and could provide a simple practical solution to eliminate global pro-vitamin A deficiency. Oil palm is a perennial, evergreen tree adapted to cultivation in biodiversity rich equatorial land areas. The growing demand for the palm oil threatens the future of the rain forests and has a large negative impact on biodiversity. Plant science faces three major challenges to make oil palm the key element of building the future sustainable world. The global average yield of 3.5 tons of oil per hectare (t) should be raised to the full yield potential estimated at 11–18t. The tree architecture must be changed to lower labor intensity and improve mechanization of the harvest. Oil composition should be tailored to the evolving needs of the food, oleochemical and fuel industries. The release of the oil palm reference genome sequence in 2013 was the key step toward this goal. The molecular bases of agronomically important traits can be and are beginning to be understood at the single base pair resolution, enabling gene-centered breeding and engineering of this remarkable crop.

## Introduction

No human activity has altered the face of the planet more than agriculture (Foley et al., 2005) that is one of the principal causes of biodiversity loss (Green et al., 2005). The increase of the global agricultural production is thought to happen in tropical countries where cropland expanded by approximately 48,000 km^2^ per year from 1999 to 2008 (Phalan et al., 2013). Cropland development is the most controversial in tropics, because they support the high species richness and endemism, and have large projected increases in demand for food from human populations growing in size and wealth (Laurance et al., 2014). Analysis of crop distribution and expansion in 128 tropical countries showed that overall expansion of annual crops has been more rapid and more widespread than expansion of perennial crops, and has occurred across much of South America, Africa, and tropical Asia. Crops that expanded most during 1999–2008 were soybeans, maize, paddy rice, and sorghum, in that order (Phalan et al., 2013). Soybean expansion is further recognized as a major cause of biodiversity loss in the Brazilian Cerrado savannas (Fearnside, 2001).

Individual crops differ in their biodiversity impacts, depending on how and where they are cultivated. Coffee covers a relatively small area, but tends to replace habitats of particularly high biodiversity value (Phalan et al., 2013). Oil palm fruit is only fifth on a list of biodiversity threats (Phalan et al., 2013), nevertheless “Few developments generate as much controversy as the rapid expansion of oil palm into forest-rich developing countries such as Indonesia” (Sheil et al., 2009). Why some crops have received relatively little attention from conservationists is a matter of debate, yet the negative impacts of the South-East Asian oil palm industry on biodiversity, and on orangutans in particular, have been well documented and publicized (Fitzherbert et al., 2008). In regard of the great apes habitat destruction, similar concerns are also expressed about the wildlife habitat conversion by the oil palm plantations in Africa (Wich et al., 2014), where about two million hectares are likely to be converted for oil palm cultivation (Murphy, 2014). Species diversity, density and biomass of invertebrate communities is estimated to suffer at least 45% decreases from land-use transformation of tropical forests to oil palm plantations (Barnes et al., 2014). Furthermore, as a substitute for reforestation, the native biodiversity of oil palm plantations is far lower than that of rubber tree plantations, which are the primary current threat to the rain forests in Cambodia (Fitzherbert et al., 2008). On the other hand, oil palm was found most sustainable with respect to the maintenance of soil quality, net energy production and greenhouse gas emissions, when biodiversity loss due to oil palm expansion was analyzed in relation to alternative crops for oil or energy, such as soybean, rapeseed, corn or sugar cane (de Vries et al., 2010). The peatland deforestation for oil palm cultivation in West Kalimantan, Indonesia have a large negative impact on greenhouse gas emissions (Carlson et al., 2012). However, global analysis of oil palm cultivation suggests that crop may encourage forest reversion and lower global emissions (Villoria et al., 2013), mainly because oil palm plantations store more carbon than alternative agricultural land uses (Sayer et al., 2012).

Deforestation and peatland degradation can be avoided when degraded lands, such as *Imperata cylindrica* grassland in Indonesia (Wicke et al., 2011) and cattle pastures in Amazon (Godar et al., 2014; Villela et al., 2014) are used for oil palm cultivation; a solution embraced by environmentalist and policy makers (World Resources Institute^1^). Even Greenpeace admits that “good palm oil” is acceptable if policy makers: (1) put an end to deforestation; (2) introduce peatland restoration policies; (3) support small-holder farms and (4) involve local communities in palm oil business^2^. To reduce the environmental footprint of oil palm, The Roundtable for Sustainable Palm Oil (RSPO) has been established in 2004^3^. The RSPO is a non-profit association that brings together palm oil producers, processors and traders, consumer goods manufacturers, retailers, banks and investors, as well as environmental and social non-governmental organizations (NGOs) to develop and implement a global standard for sustainable palm oil in order to produce Certified Sustainable Palm Oil (CSPO). On the other hand, smallholder farmers have difficulties to meet CSPO criteria (Murphy, 2014).

Why this global integration is so important? Oil palm cultivation is one of the most profitable land uses in the humid tropics (Sayer et al., 2012). In the state of Pará, Brazil for example, the average annual monetary return on investment is US$ 2000 per hectare (Villela et al., 2014). With further 32 million hectares of degraded land suitable for oil palm cultivation (Ramalho-Filho et al., 2010) crop has a potential to evolve into a multibillion dollar business in Brazil. The crop is often considered as an industrial crop, but in many areas it is a valuable smallholder crop (Feintrenie et al., 2010). Globally, three million smallholders live from oil palm cultivation. The share of palm oil production by small, family-owned, estates is 30% worldwide and reaches 80% in Nigeria, Africa’s largest producer (Morcillo et al., 2013). Government policies in Malaysia, Indonesia and Brazil favor smallholder involvement in the oil palm industry. Indonesia has a target of 40% production coming from smallholders, who supply the oil mills. In Indonesia, 25 million people livelihood depends one way or another on oil palm production (Murphy, 2014). Thus, oil palm cultivation alleviates poverty and with right governmental policies could transform livelihood of millions of people (Sayer et al., 2012).

Oil palm (*Elaeis guineensis*, Jacq.) is by far the most productive oil crop and alone is capable to fulfill the large and growing world demand for vegetable oils that is estimated to reach 240 million tons by 2050 (Corley, 2009). Per hectare of cropland, oil palm plantations give 3–8 times more oil than any other temperate or tropical oil crop. In 2012 for instance, 56.2 million tons of palm oil were produced on 17.24 million hectares. Only 23.6 million tons of oil were extracted from rapeseed grown on 36.4 million hectares^4^. On November 2014, palm oil was valued as a vegetable oil with the lowest production costs by the international commodities markets^5^, e.g., US$ 700 and US$ 850 per metric ton of oil palm and rapeseed oil, respectively. The oil palm cultivation however, is labor intensive. As the labor costs increase, overseas workers are often providing cheap labor force. Half a million Indonesian workers are now being recruited to Malaysian oil palm plantations. Manpower shortfall resulted in 15% losses of fruits in East Malaysia, whilst the lack of good field training is a further contributing factor for production losses (Murphy, 2014). It is a challenge for palm breeders to alter tree architecture in such a way as to lower the labor intensity of the crop and to facilitate mechanization of the harvest.

Oil palm is a C3 photosynthesis, evergreen tropical perennial tree (Corley and Tinker, 2003). Adult palms planted at optimal density of 130–150 trees per hectare are on a relative steady state in terms of canopy development and have a large leaf area index between 4 and 5, leading to a light interception efficiency close to one (Pallas et al., 2013). Unlike other studied angiosperms, oil palm does not regulate photosynthesis to adjust source–sink imbalances (Legros et al., 2009), instead photosynthates are converted into a reserve pool of non-structural carbohydrates (NSC) mainly located in the tree trunk as glucose and starch. The main physiological function of transitory NSC storage is to balance sink and source fluctuations within a day to seasons. The NSC reserve pool in oil palm is so large that it can theoretically sustain tree growth for 7 months, and most importantly to maintain fruit growth and energetically costly oil biosynthesis at fruit maturation, regardless of cloud cover or periodical suboptimal growth conditions (Legros et al., 2009). In contrast, intercepted solar radiation during seed filling is the rate limiting in an annual oil crop sunflower, and determines weight per seed and oil concentration (Aguirrezábal et al., 2003). Thus, unusual characteristics of source-sink interactions, combined with high efficiency of solar radiation interception and continuous year-round fruit production are likely ecophysiological traits that determine superior productivity of oil palm (Basri Wahid et al., 2005).

Plant scientists commonly argue that finding solutions for increasing crop yield potential, e.g., doubling yield by improving photosynthesis efficiency, and closing the yield gap will satisfy food demand by the growing human population that is estimated to reach 9–10 billion by the year 2050. The challenge for oil palm planters will be to close the yield gap between the average plantation output at present 3.5t, compared to some best known varieties that in favorable agro-climatic conditions produce up to 9–12t (Murphy, 2009). However, the intensification of land use appears to result in further biodiversity loss, the so-called trade-offs between biodiversity value and yield (Phalan et al., 2011, 2014). High-yield oil palm expansion spares land at the expense of forests in the Peruvian Amazon (Gutiérrez-Vélez et al., 2011). Thus, increase in yield potential of oil palm crop is a necessary, but not sufficient requirement for the sustainable future of tropical forests.

The aim of this review is to acquaint the reader with natural diversity of oil palm species and how it can be used to increase productivity of the future oil palm plantations. We will begin by covering basic facts about oil palm biology and cultivation, followed by a brief review of germplasm and genomic resources, and how these have been used for gene discovery and breeding purposes. The potential of other oleaginous palms to lower environmental impact of oil palm cultivation is emphasized.

## Oil Palm Biogeography, Biology and Cultivation

The genus *Elaeis* of the monocotyledonous palm family *Arecaceae* was formally introduced into botanical classification in 1763 by Nicholas Joseph Jacquin, who described *Elaeis guineensis*, known as African oil palm. The Greek word “ελαιoυ”—oil, transliterated “elaion,” gave the genus name (Hartley, 1977). The genus comprises two taxonomically well-defined species, the second is American oil palm, *E. oleifera*. The well-known, phylogenetically closest relative of oil palms are coconut palms, *Cocos nucifera*, which are also vegetable oil producing crop.

### African Oil Palm

The world-wide grown crop is African oil palm naturally abundant in all the African rain forests. Both climate, and humans shaped modern biogeographic distribution. The late Holocene phase of dramatic forest decline, around 2500 years ago was favorable for the expansion of this sun-loving, pioneering species (Maley and Chepstow-Lusty, 2001). The oleaginous properties of the fruits were important in the subsistence economy in Africa for the past 5000 years (Sowunmi, 1999).

Analysis of the species natural genetic diversity suggests that wild populations could be separated into three groups located at the extreme west of Africa, equatorial Africa and on Madagascar Island. The highest allelic diversity was found among Nigerian palm populations, indicating the possible center of origin (Bakoumé et al., 2015). Semi-wild feral populations of African oil palm found in a Brazilian state Bahia are very similar to palms from Nigeria and were most likely established during the period of slave trade (Bakoumé et al., 2015).

Thus, the majority of wild oil palm populations inhabit tropical lowlands with the average annual rainfall of about 1780–2280 mm and temperature ranging from 24 to 30°C. Accordingly, varieties standard in cultivation are usually sensitive to water deficit. The atmospheric humidity also strongly influences oil palm photosynthetic capacity. Low air humidity restricts stomatal opening and CO_2_ uptake (Smith, 1989). Another ecophysiological characteristic that limits the latitude and altitude ranges of cultivation is cold sensitivity. The growth of common oil palm varieties is suppressed at ambient temperatures below 15°C (Corley and Tinker, 2003). Oil palm can be cultivated on a broad range of soils (Corley and Tinker, 2003).

African oil palm trees can reach 15–18 meters in height, up to 30 meters in a dense forest. It is believed that some palm groves are more than 200 years old (Corley and Tinker, 2003). The leaves could be 8 meters in length. It takes about 2 years for the first leaf primordia to reach the fully expanded stage. To achieve maximal yield on commercial plantations, the leaf length is a critical trait that determines tree planting density.

Oil palms are monoecious species that produce unisexual male and female inflorescences in an alternating cycle. Such “temporal dioecism” results in allogamous reproduction by cross-pollination (Adam et al., 2011). Inflorescences are enclosed during their development by spathe, a large bract, which is ruptured just before flower maturity is reached. Both genetic and environmental factors influence inflorescence sex determination. Reduced photosynthesis due to defoliation, or high density planting, for example, promotes male inflorescence development. This observation was critical for the development of industrial scale seed production by controlled pollination (Durand-Gasselin et al., 1999). Breeding for higher productivity result in varieties that produce larger number of female inflorescences and shortage of pollen. Thus, pollination efficiency has a large impact on yield of the crop.

Whilst wind pollination could occur, maximal pollination efficiency depends on insects. The main pollinators are weevils, a type of beetles, of the genus *Elaeidobius* spp., in particular *E. kamerunicus*. Weevils complete the entire life cycle by feeding on the palm male flowers and male inflorescence tissues. The aniseed scent that is the same between female and male inflorescences, is attributed to the emission of methyl chavicol (Lajis et al., 1985), that attracts *E. kamerunicus* (Hussein et al., 1989). In a search for the male inflorescences, weevils visit female flowers, depositing pollen grains by accident (Tandon et al., 2001). To achieve maximal pollination efficiency, *E. kamerunicus* were introduced on plantations both in South-East Asia, and Latin America (Corley and Tinker, 2003).

The oil palm fruit is a sessile drupe. Fruits grow in large bunches and mature in 5–6 months after pollination. Oil palm accessions show great variation in fruit shape and size (Corley and Tinker, 2003). The pericarp of the oil palm fruit is subdivided into the outer layer exocarp, fleshy mesocarp, and endocarp that in oil palm, is called shell. Shell encases the seed or kernel, i.e., embryo and endosperm. The crude palm oil and kernel palm oil are extracted from mesocarp and kernel, respectively.

In wild type palms, endocarp thickness varies from 2 to 8 mm in between different accessions (Corley and Tinker, 2003). Endocarp development depends on the major effect *SHELL* gene. *SHELL* mutant alleles with co-dominant monogenic inheritance characterize the so-called *pisifera* palms (*sh/sh*) that produce shell-less fruits (Beirnaert and Vanderweyen, 1941). Wild type *Sh/Sh dura* palms develop fruits with thick endocarp. Intraspecific heterozygous (*Sh/sh*) hybrids, known as *tenera* palms, have thinner shells surrounded by a distinct fiber ring (Beirnaert and Vanderweyen, 1941). The shell thickness has major effect on oil content, with *teneras* having 30% more mesocarp and respectively 30% greater oil content in bunches than *duras* (Corley and Tinker, 2003). Owing to their higher oil yields, *tenera* palms were selected already by the pre-colonial cultures in West Africa (Devuyst, 1953).

**FIGURE 1:**
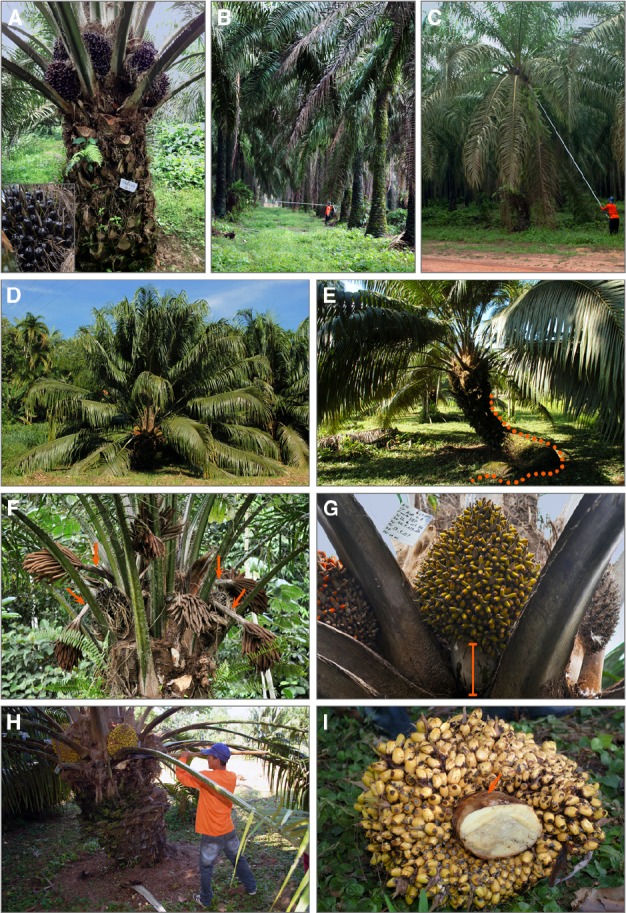
***E. guineensis* and *E. oleifera.* (A)** Commercial African *tenera* oil palm from a cross *dura* Deli × *pisifera* Nigeria. The tree is 5-years-old and has *nigrescens* fruit type (inset). **(B)** 26-years-old plantation of *tenera* palms (Deli × Ghana). Trees are 7–8 meters tall. **(C)** Fruit bunch harvest. A worker is using a knife to cut off bunches from a tree on a plantation shown in **(B)**. Bunch ripeness is assessed by the presence on the ground of the shed-off fruits. **(D)** *E. oleifera*. The wild tree that is more than 30-years-old (Manicoré, Amazonas, Brazil). **(E)** Walking palm. The same tree as in **(D)** photographed at different angle to illustrate procumbent trunk that is outlined by the dotted line. **(F)** African oil palm male inflorescences. This individual has particularly long male inflorescence stalks that are pointed with arrows. **(G)** Fruit bunch stalk. The 3-months-old fruit bunch of the American oil palm is shown. Bar indicates short stalk. **(H)** Collection of *E. oleifera* bunch. Harvesting oil palm requires skilled workers, even when trees are not very tall. **(I)** Thickness of the bunch stalk. The cut-off bunch from a tree in **(H)**. Cutting through the bunch stalk (orange arrow) composed of a very fibrous tissue, requires physical strength.

African oil palms have different coloring of exocarp, producing either *nigrescens* or *virescens* fruit types. Type *nigrescens* accumulate large amounts of anthocyanins, which accounts for the deep violet to black color at the fruit apex (Figure 1A). When unripe, *virescens* fruits are green turning orange due to accumulation of carotenoids and chlorophyll degradation in relation to ripening. Five spontaneous dominant mutant alleles in a *VIRESCENS* gene abolish anthocyanin synthesis, which explains the *virescens* fruit type (Singh et al., 2014). Fruit phenotype *nigrescens* is a likely wild type. The occurrence of *virescens* palms is usually less than 1%, however, in some Congolese oil palm populations up to 50% of trees will produce *virescens* fruits. Artificial selection by local communities has driven the persistence of the newly arising mutations (Zeven, 1972).

In most of angiosperms, flowers and immature fruitlets are naturally thinned by organ abscission in response to nutritional status. This phenomenon is negligible in oil palm that only shed ripe fruits (Roongsattham et al., 2012). It is plausible that low abscission of flowers and immature fruitlets contributes to the exceptional oil palm productivity. Ripe fruit shedding is a main indicator whether bunch is ready to be harvested (Corley and Tinker, 2003), on the other hand, shedding is one of the causes of losses at harvesting (Osborne et al., 1992).

The stalk of fruit bunches is short and thick in oil palms (Figures 1G,I). The stalks of male inflorescences are longer (Figure 1F). Cutting fruit bunches off the trees is a laborious process (Figures 1B,C,H), thus short and thick stalks are the traits that limit harvest mechanization (Le Guen et al., 1990).

To achieve synchronous germination of commercially produced seeds, combined temperature and humidity treatments are required to break the dormancy. Each germinated seed is maintained in a pre-nursery for 4–5 months till a plantlet reaches a four-leaf stage, after which young palms are grown for about a year in a nursery before they are transferred to the field. Establishment of leguminous cover prior planting prevents soil erosion and surface run-off, improves soil structure and palm root development, increases the response to mineral fertilizer, and reduces the danger of micronutrient deficiencies (Corley and Tinker, 2003).

### American Oil Palm

*Elaeis oleifera* (Kunth, Cortés) is known as the American oil palm. Species is native to and broadly dispersed in Central America and northern regions of South America. Small and dense *E. oleifera* populations grow along the riverbanks, tolerating well both shade, and flooding, indicating a broader environmental adaptability compared to the African oil palm (Corley and Tinker, 2003). Judging by the higher morphological variation of the trees, the region occupied by Colombia, Suriname, and North-West Brazil is thought to be the species center of origin (Meunier, 1975; Ooi et al., 1981). In the Amazon River Basin that is considered a center of secondary diversification (Meunier, 1975; Barcelos et al., 2002), many *E. oleifera* populations are found on Amazonian Dark Earths, Terra Preta de ĺndio in Portuguese. Amazonian Dark Earths were formed in the past by pre-Columbian populations and are highly sustained fertile soils supported by microbial communities that differ from those extant in adjacent soils (Lima et al., 2014). In spite of this association of palms with human habitation, there are no historical indications of artificial selection for improved yield that in *E. oleifera* remains significantly lower compared to the African oil palms. Oil to bunch ratio of *E. oleifera* is about 5%, as compared to 25% in *E. guineensis teneras* (Barcelos, 1998b).

**FIGURE 2:**
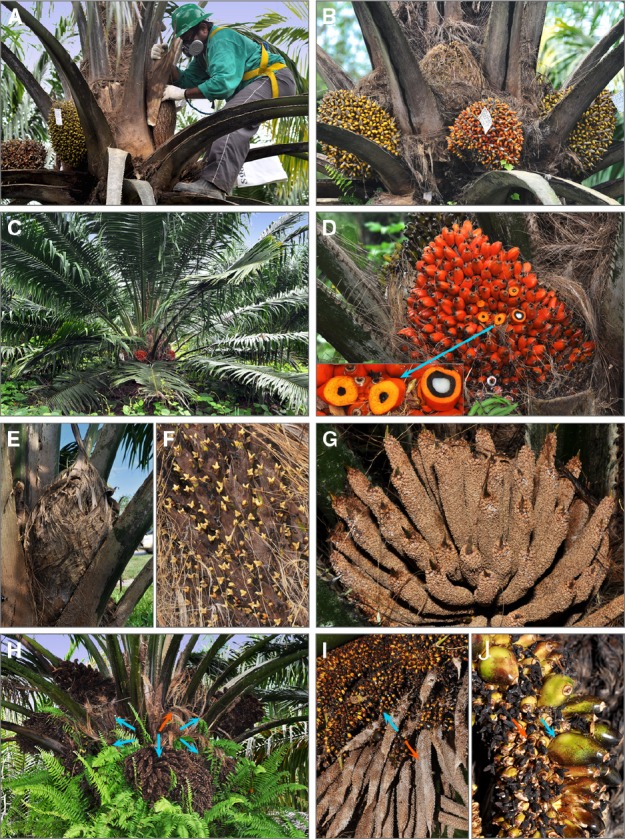
***E. oleifera × E. guineensis* interspecific hybrids at EMBRAPA breeding station Rio Urubu (Amazonas, Brazil). (A)** Controlled pollination. Worker is removing the spathe on elite *E. oleifera* tree that is three meters tall. He wears safety gear to climb the tree, protective mask and gloves. In commercial seed production, female inflorescences are bagged 1 week before anthesis. **(B)** Bunch ripening. Three bunches at different times after pollination are visible. As fruit mature, the fruits change color from green to orange. The youngest female inflorescences were not used in pollination, those bunches are still enclosed in spathe, which is an *E. oleifera* species-specific characteristic. **(C)** F1 interspecific hybrid on commercial plantation. Tree is 4 years old. It has about twice shorter stem than African oil palm trees of the same age. Orange mature bunches are easily spotted within the tree crown. **(D)** Parthenocarpic fruits in mature bunch of the F1 interspecific hybrid. Insert: a few fruits were transversely cut. The fruit to the right has normal seed. White colored endosperm tissue of the kernel is surrounded by the black endocarp, mesocarp is orange. Parthenocarpic fruits have residual black endocarp and no kernel. **(E)** Female inflorescence of F1 hybrid at anthesis. Inflorescence is still enclosed in spathe, which is similar to the parent *E. oleifera.* **(F)** Female flowers. The same inflorescence as in **(E)**. Spathe was removed to show female flowers at anthesis. Three-lobed flower stigmas are yellowish in color and receptive for pollen. **(G)** Male inflorescence of F1 hybrid at anthesis. The overall morphology and flower identity are normal. **(H)** Andromorphic inflorescences. This 15-years-old tree developed five fully andromorphic inflorescences pointed with blue arrows and a single male inflorescence (behind the leaf rachises to the right). Orange arrow points a fruit bunch. **(I)** Partial andromorphy. Individual andromorphic inflorescence can show different proportions of male flowers (orange arrow) and female flowers (blue arrow). **(J)** Fruitlets from andromorphic inflorescence. Some fruitlets abort after anthesis, died dry flower pistils are black (orange arrow), other develop parthenocarpically (blue arrow). Andromorphic inflorescences in **(H)** are full of developing fruitlets.

A distinguishing feature of *E. oleifera* is a much shorter, often procumbent trunk, a trait from which species are also known as a walking palm (Figures 1D,E). After procumbence, the basal part of the plant dies whilst adventitious roots sprouting from the part in contact with soil allow plant to restart growth. The high proportion of parthenocarpic fruits that may constitute up to 90% of the total is another striking characteristic of the *E. oleifera* fruit bunches as compared to the African species. Parthenocarpic fruits often abort, contributing to poor yield. Immature fruits are green turning orange at maturity, which resembles *virescens* African oil palms (Figures 2A,B). *E. oleifera* leaves have a different from *E. guineensis* positioning of leaflets (Corley and Tinker, 2003). *E. oleifera* pollination depends on insects. However, the profile of volatiles emitted by the inflorescences at anthesis is different and species do not synthesize methyl chavicol (Gomes, 2011).

### Interspecies Oil Palm Hybrids

African and American oil palm species are sexually compatible (Hardon and Tan, 1969). F1 hybrids show vegetative vigor and mid-parent stem growth increment (Corley and Tinker, 2003). *E. oleifera* leaf morphology and parthenocarpic fruit development (Figure 2D) behave as dominant traits. F1 hybrids from crosses between some *E. oleifera* accessions and *nigrescens E. guineensis* parents, have *virescens* phenotype of fruits that are bright orange at maturity (Figures 2C,D).

To achieve reasonable yield from F1 hybrids, assisted pollination is required, i.e., one worker to pollinate 10–20 hectares of a plantation. In relation to the accession of the *E. oleifera* and *E. guineensis* parents, a number of developmental abnormalities could contribute to lower F1 hybrid fertility, including lower pollen yield, poor pollen germination, poor anther dehiscence (Corley and Tinker, 2003); lower emission of volatiles by the inflorescences at anthesis (Gomes, 2011), which is a likely cause of poor attractiveness for *E. kamerunicus* (Tan, 1985). The spathe encasing female inflorescence at anthesis could present a mechanical barrier for pollinating insects (Figures 2B,E,F). Of interest for developmental biologists are the andromorphic inflorescences, a trait that reduce pollen production and characterize some F1 hybrid combinations (Figures 2G–J). Whether allopolyploidization will alleviate, or exacerbate fertility problems of the interspecific oil palm hybrids is unknown.

It is possible that some of the F1 hybrid developmental abnormalities and dominance-recessiveness gene interactions can be attributed to the non-additive gene expression that is typical for interspecific hybrids (Chen, 2013). In a view of oil palm genomics development, it is tempting to consider ion beam deletion mutagenesis (Ishikawa et al., 2012) as an experimental tool to identify the molecular basis of the traits that distinguish oil palm species and their hybrids.

## Natural Variation for Oil Palm Improvement

The large scale establishment of Congolese and South-East Asian commercial plantations in 1910–1920s, was quickly followed by the research on the crop improvement by selection and breeding (Corley and Tinker, 2003). Oil palm breeding was influenced by maize breeding that relies on development of inbred parental lines to produce homogeneous F1 hybrids. Accordingly, reciprocal recurrent selection and family-individual selections methods were commonly chosen by oil palm breeders for the development of parental lines that are used in commercial F1 hybrid seed production. Ten percent productivity gains per decade were reported by the French and Malaysian breeding programs (Corley and Tinker, 2003).

The exploitation of the superior oil content of the *teneras* began in 1930s on Congolese plantations, which led Beirnaert to explain *dura*, *pisifera*, and *tenera* phenotypes by the co-dominant monogenic inheritance of the *shell* (*sh*) mutant allele (Beirnaert and Vanderweyen, 1941). On Malaysian plantations, the cultivation of *teneras* (*Sh/sh*) produced by controlled pollination of *duras* (*Sh/Sh*) with pollen of *pisifera* palms (*sh/sh*) took off in 1956. Most of commercial seeds today are intraspecific *dura* × *pisifera* (D × P) hybrids (Corley and Tinker, 2003).

Oil palm breeders use “breeding populations of restricted origin” (BPRO) that can be traced back to distinct, often small groups of wild or unimproved ancestral palms (Rosenquist, 1986). For example, Deli *duras* are used today as the mothers for almost all commercial *teneras* seed production. The Deli *dura* palms can be traced to four individuals planted in the Bogor Botanical Gardens (Java, Indonesia) in 1848 (Kushairi and Rajanaidu, 2000; Cochard et al., 2009). Most commonly used *pisiferas* descend from limited number of origins, as well. A single Django *tenera* palm from Congo, gave rise to the AVROS (Algemeene Vereniging van Rubber Planters ter Oostkust van Sumatra, now Indonesian Oil Palm Research Institute—IOPRI^6^) *pisiferas* widely used for seed production in Indonesia, Malaysia, Papua New Guinea, and Costa Rica (Rajanaidu et al., 2000; Corley and Tinker, 2003). Thus, commercial *teneras* have a narrow genetic base due to the restricted number of ancestral progenitors (Hardon, 1968; Ooi and Rajanaidu, 1979).

Many breeders realized the need for new material of sufficient genetic variability for future progress. Thus, the major task of the oil palm research institutions was adequate characterization of the natural diversity either by prospection of *in situ* natural populations, or by the establishment of *ex situ* germplasm collections. Beginning in 1950s, tens of thousands of palms from *in situ* natural populations have been screened at low cost to isolate just a few elite individuals that were then used in breeding programs. This strategy was largely adopted by the Institut de Recherches pour les Huiles et Oléagineux (IHRO), presently CIRAD^7^ (Meunier, 1969). Since 1970s, to capture broader spectra of natural variation, and to ensure protection of the trees from destruction, Palm Oil Research Institute of Malaysia (PORIM), presently Malaysian Palm Oil Board (MPOB)^8^ engaged in random sampling of palms from wild populations to establish *ex situ* germplasm collection of 1467 accessions (Rajanaidu, 1994). This strategy is more expensive than *in situ* prospection. MPOB Nigerian collection alone occupies 200 hectares. However, *ex situ* approach allows more thorough characterization of the traits of interest, and provides guarantees for preservation of traits that might become of interest in the future (Rajanaidu et al., 2000; Paterson et al., 2013). The fate of *in situ* germplasm is unpredictable, they can be either destroyed, or replanted. In the survey organized by the Food and Agriculture Organization of the United Nations, 29 participating institutions reported a total of 21103 oil palm accessions (F.A.O, 2010)^9^.

Phenotypic screens of the *E. guineensis* germplasm collections conducted by the main oil palm research centers MPOB (Malaysia), CIRAD (France), IOPRI (Indonesia), and the Empresa Brasileira de Pesquisa Agropecuária (EMBRAPA, Brazil) revealed a significant phenotypic diversity for the valuable agronomical characteristics, such as: (1) leaf petiole, rachis length, i.e., breeding for the so-called compact palms; (2) increment in the growth in height, i.e., breeding for shorter palms; (3) bunch number, weight and production, i.e., oil yield; (4) fresh fruit bunch and crude palm oil yield; (5) total and vegetative dry matter production; (6) fruit and kernel size, i.e., fatty acid (FA) profile; (7) fruit shell thickness; (8) Fusarium wilt disease tolerance; (9) FA composition and iodine value; (10) carotene and vitamin E contents; (11) lipase activity; (12) *in vitro* regeneration potential; (13) drought and cold tolerance (Rajanaidu et al., 2000; Corley and Tinker, 2003). Based on the phenotypic data, selected accessions have been subsequently used to develop improved *dura* and *pisifera* fruit type parental palm varieties.

Oil palm breeders became interested in *E. oleifera* agronomic potential at the beginning of the last century. In 1920s *E. oleifera* was introduced in Africa and in 1950s to Asia. However, it is only in the last 30–40 years that *E. oleifera* natural populations have been thoroughly sampled to establish *ex situ* germplasm collections in Malaysia, Ivory Coast, Costa Rica and Brazil (Meunier, 1975; Escobar, 1981; Ooi et al., 1981; Rajanaidu, 1986; Barcelos et al., 1999, 2002). FAO Database registered 506 accessions, of which 244 are maintained by the EMBRAPA on a breeding research station^10^ located in the municipality of Rio Preto da Eva, state Amazonas, Brazil.

American oil palm is a source of many economically valuable traits, of which most important are (1) slow height increment, which simplify harvest (Corley and Tinker, 2003); (2) higher proportion of desaturated FAs in palm oil (Montoya et al., 2014); (3) lower lipase activity in mature fruit mesocarp, extending a period between harvest and fruit processing (Sambanthamurthi et al., 1995; Cadena et al., 2013); (4) higher vitamins A and E contents, improving oil nutritional value (Rajanaidu et al., 2000) and (5) broader environmental adaptability (Barcelos, 1986, 1998b). In addition, American oil palm is also more resistant to several diseases (Corley and Tinker, 2003), including bud-rot caused by *Phytophthora palmivora* and *Fusarium* wilt (Barcelos, 1986).

Hybrids between *E. guineensis* × *E. oleifera* excited much interest, because of the slower growth and higher desaturation of palm oil (Corley and Tinker, 2003). Interest in interspecific hybrids further increased with a recognition of their resistance to fatal yellowing disease that is a major threat to oil palm cultivation in Latin America, a discovery that led to the first commercial plantations in 1980s. In the study published in 1995, Amblard et al. (1995) analyzed 429 hybrid progenies obtained by crossing *E. oleifera* and *E. guineensis* of different origin. Bunch and oil production in the best inter-origin combinations reached 85 and 78% of the average values for the commercial oil palm cultivars, respectively. Some selected hybrids have oil productivity as high as commercial *teneras*, but only with assisted pollination because of the serious fertility problems. Seeds of high yielding interspecific hybrids are produced by CIRAD/PalmElit^11^ (COARI hybrids) and EMBRAPA/Dendê do Pará S.A.^12^ (MANICORÉ hybrids), using wild *E. oleifera* palms indigenous to the Coari and Manicoré municipalities in the Amazon river basin.

## Genomics for Oil Palm Improvement

Oil palm is a diploid (2n = 32) with an estimated genome size of 1.8-gigabases (Gb). A total of 1.535 Gb of the *E. guineensis* (AVROS, *pisifera* fruit form) reference genome assembly were released to public in 2013 (Singh et al., 2013b) and is freely available^13^. For comparative purposes, the genome the American oil palm was sequenced. Most of the reference genome is represented by segmental duplications, and not triplications, indicating that oil palm is a paleotetraploid. Analysis of conserved gene order revealed that the duplications were retained in *E. oleifera*, so that segmental duplications pre-dated the divergence of the African and American oil palms. On the other hand, 57% of the 1.8-Gb *E. guineensis* genome comprises repetitive elements of which 47% were uncharacterized previously, with 73% absent from *E. oleifera* genome, indicating extensive molecular speciation that might account for the fertility problems of interspecific oil palm hybrids.

Genome sequence and transcriptome data from 30 tissue types were used to predict at least 34,802 genes (Singh et al., 2013b). De novo assembly from RNA-seq data resulted in 51,452 oil palm unigenes (Lei et al., 2014). To characterize the genic regions in a greater detail, the methylation filtered libraries of the African and American oil palm species were sequenced (Low et al., 2014). Sequence analysis revealed single nucleotide polymorphisms (SNP) at densities 2.30 and 2.83 per 100 bp for *E. guineensis* and *E. oleifera*, respectively.

For a perennial tree that flowers only 2–3 years after seed germination, breeding oil palm requires 10–19 years per cycle of phenotypic selection (Wong and Bernardo, 2008). Molecular breeding uses genetic markers linked to the traits of choice for earliest pre-selection of desired phenotypes and has a potential to greatly shorten the breeding cycle, reducing costs. The data of (Low et al., 2014) were used to generate a final set of 4,451 SNPs that were selected for developing a customized oil palm specific SNP array (OPSNP3) printed on the Infinium HD iSelect BeadChips platform (Ting et al., 2014). Genotyping across 199 palms from two separate mapping F1 hybrid populations, e.g., *E. oleifera* × *E. guineensis* interspecific cross and a *dura* × *pisifera* intraspecific cross took less than 3 months (Ting et al., 2014) and greatly improved marker density and genome coverage in comparison to the first reference maps based on AFLP and SSR markers (Barcelos, 1998a; Barcelos et al., 2002; Singh et al., 2009; Billotte et al., 2010; Ting et al., 2013). Refined genetic maps combined with careful phenotyping of trees are likely to facilitate mapping and identification of molecular bases of both monogenic, and quantitative trait loci (QTL) that underpin major agricultural traits of interest (Yang et al., 2014).

## Increasing Oil Palm Productivity

The immediate impact of the oil palm genome sequence was identification of the *SHELL* gene (Singh et al., 2013a) that was shown to encode a MADS-box transcription factor homologous to the *Arabidopsis* ovule identity and seed development regulator SEEDSTICK. Two different amino-acid substitution mutations in a dimerization and DNA-binding domain of the SHELL protein occur in the *sh^MPOB^* and *sh^AVROS^* spontaneous mutant alleles. Mutant proteins are likely to act as *trans*-dominant negative isoforms, which explains the co-dominant phenotype in *tenera* palms. Molecular markers for *SHELL* gene alleles could be used to distinguish *dura*, *tenera*, and *pisifera* plants in the nursery long before they are planted in a field. Nursery stage screening can eliminate erroneous planting of *dura* palms and control the precision of hybrid seed production. Marker-assisted introgression of the *SHELL* gene alleles on different genetic backgrounds could accelerate construction of new *dura* and *pisifera* palms.

It will be important to understand whether *sh^MPOB^*, *sh^AVROS^* alleles or similar *trans*-dominant alleles constructed by protein engineering will show a dosage effect that could further increase the oil yield in palms, which have genotype *Sh/sh/sh*, for example. The effect of the *SHELL* gene variation on the mesocarp yield of other commercially useful palm species, such as date (*Phoenix dactylifera*), açaí (*Euterpe oleracea*), peach (*Bactris gasipaes*) palms, can be tested.

Further yield enhancements were brought by breeding *dura* and *pisifera* parents for the higher ratio of female inflorescences; bunch weight; oil to bunch ratio; oil recovery and earlier flowering. Donors of such traits are breeding accessions of Deli palms that have high yield; AVROS lines characterized by precocity, high yields and growth vigor; Ekona palms that have high oil to bunch ratio and earlier flowering Yangambi palms (Alvarado et al., 2010).

Best *dura* × *pisifera* combination have 28–32% oil to bunch ratio and can produce annually up to 10t, e.g., (i) CIRAD Deli × Yangambi (PalmElit^14^); (ii) Evolution (Alvarado et al., 2010)^15^, crosses of Deli *dura* with composite *pisifera* carrying traits introgressed from several oil palm populations.

Potential yield of hypothetical oil palm genotypes that combine physiologically plausible attributes is being estimated at about 18.5t (Corley, 1998), which is almost a double of the best varieties on the oil palm seed market. Site yield potential varies amongst individuals of the same descent, in part due to residual genetic variation in parental lines. Exceptionally high yields of 12–13t could be anticipated if some well performing individual trees could be multiplied (Sharma and Tan, 1997). Oil palms do not branch unless terminal single vegetative shoot apical meristem is damaged. Thus, to produce planting material from high yield elite individuals, the only practical way of vegetative propagation is *in vitro* clonal propagation, micropropagation (Corley and Tinker, 2003). Oil palm micropropagation remains an inefficient, lengthy process, however. Most genotypes are recalcitrant in tissue culture, which requires empirical tests of numerous media formulas. Introgression into elite varieties the superior somatic embryogenesis capacity known for some palm accessions is considered a partial solution to the problem (Ting et al., 2013). Inducible versions of genes that promote somatic embryogenesis is a worthwhile approach (Heidmann et al., 2011) that has not been tested with oil palm.

The alternative to micropropagation of oil palm can be a reverse breeding, which is a plant breeding technique to produce parental lines for any heterozygous plant (Dirks et al., 2009). The suppression of meiotic crossovers and transmission of non-recombinant chromosomes to haploid gametes is a key to reverse breeding. Gametes are subsequently regenerated as doubled-haploid offspring among which the parental lines are selected (Wijnker et al., 2014). Thanks to the knowledge of the oil palm gene space (Low et al., 2014), meiosis regulators, such as DMC1orthologs (Wijnker et al., 2012) can be identified and then controlled. The frequencies of spontaneous haploids in seed progeny are very low, 1,100 haploids among 60 million seedlings (Dunwell et al., 2010). Cultures of oil palm microspores have not yielded doubled-haploids so far (Corley and Tinker, 2003). The totipotency of the male gametophyte is thought to be negatively regulated by a histone deacetylase-dependent mechanism, which is affected by the stress treatments, such as cold or heat shock that are used to induce haploid embryo development in culture (Li et al., 2014). Two percent of oil palm microspores exposed to the low temperature and starvation stress initiated cell division and formed embryoids (Indrianto et al., 2014). It is likely that inhibitors of histone deacetylases, trichostatin A for example (Li et al., 2014), will further increase the efficiency of oil palm microspore reprogramming to somatic embryogenesis. Genotyping doubled-haploids using SNP arrays (Ting et al., 2014) could then enable reverse breeding of oil palms.

## Yield Gap Caused by the Diseases

Two major diseases threaten oil palm industry. In South-East Asia, the basal stem rot disease that is caused by the white rot fungi of the genus *Ganoderma* spp. is the major problem (Paterson, 2007; Rees et al., 2009). Nearly 60% of plantations in Malaysia reported the diseased trees. The basal stem rot is lethal, infected plants stop producing fruit and eventually die. The average tree mortality rate of 3,7% is equivalent to losses of US$ 570 million per year (Mohammed et al., 2014). White rot fungi are characterized as facultative saprophytes, which are generally difficult to control. There are no good sources of natural genetic disease resistance neither amongst African, nor American oil palm accessions (Durand-Gasselin et al., 2005). The research efforts have focused on a more detailed understanding of the molecular defense responses in those plant-pathogen interactions, with a hope to find practical solutions to control the disease (Ho and Tan, 2014). To make cellulose available, white rot fungi are capable of degrading lignin to carbon dioxide and water (Paterson, 2007). Thus, understanding lignin biosynthesis in oil palm is of interest, whilst lignin structure modification by breeding could result in genetic resistance. Candidate genes for breeding basal stem rot resistance are being looked for amongst transcripts and proteins that alter their expression patterns upon infection (Ho and Tan, 2014). Current practical solutions are biological control with *Trichoderma* spp. fungi, palm endophytes and implementation of correct agronomical and phytosanitary practices (Mohammed et al., 2014).

The mysterious and devastating disease known as a fatal yellowing (transliterated from Portuguese “amarelecimento fatal”) or lethal bud rot (“pudrición de cogollo,” Spanish) is considered a major problem for the oil palm industry in Latin America (Chinchilla, 2008). Entire estates in Panama, Colombia, Suriname, Brazil, and Ecuador were destroyed by the disease (De Franqueville, 2003). Fatal yellowing has variable symptoms, which causes considerable confusion in a research field (Corley and Tinker, 2003). In spite of numerous efforts testing candidate fungi, bacteria, phytoplasma and viroids, there is no conclusive evidence that a phytopathogen is the primary cause of the disease. Microorganisms are thought to play rather an opportunistic role in the development of the disease that is primed by certain environmental factors. Remarkably, the root system development is altered even before affected palms show symptoms in the shoot (Chinchilla, 2008). Morphological and histological study showed that contrary to healthy-looking palms, diseased palms from Ecuador and Brazil did not have roots with soft and white tips, the so-called fine root system. Only a few meristematic cells could be detected in the apical shoot and root meristems, indicating cell cycle arrest (Kastelein et al., 1990). This finding can be validated by using reporters of the entry into the M-phase of the cell cycle. Chemical screening to promote re-activation of the cell cycle in affected roots could result in a treatment of the fatal yellowing disease.

*Elaeis oleifera* shows resistance to fatal yellowing. The trait is dominant in interspecific *E. oleifera* × *E. guineensis* F1 hybrids. In spite of the lower productivity and the need for manual pollination, F1 hybrid seeds are produced on commercial scale for planting. Otherwise, we are not aware of any systematic efforts to introgress *E. oleifera* fatal yellowing genetic resistance onto African oil palm genetic background. Most probably because there is no causality link between (a)biotic factors and disease development, which makes the screening procedures unpredictable. On a contrary, ASD Costa Rica released for sale the seeds of an unusual interspecific hybrid variety AMAZON^16^, which is rather an introgression of the *E. guineensis* productivity traits onto American oil palm genetic background.

## Palm Oil Composition and Content

Along with coconut oil, crude palm oil, and particularly kernel palm oil, are some of the few highly saturated vegetable fats. On average, crude palm oil contains 44% palmitic acid (C16:0), 5% stearic acid (C18:0) and traces of myristic acid (C14:0), which together constitute half of FAs found in triacylglycerols (TAG) synthesized by the *E. guineensis* fruit mesocarp (Sambanthamurthi et al., 2000). TAG unsaturated FAs are represented by 40% of oleic acid (C18:1), 10% linoleic acid (C18:2) and traces of linolenic acid (C18:3). Food industries consume eighty percent of palm oil, also as a replacement for *trans*-FAs. Oleochemical industry manufacture soaps, detergents, lubricants, solvents, bioplastics and biodiesel from the remaining twenty percent of palm oils.

Dietary FAs play significant roles in the cause and prevention of cardiovascular disease. *Trans*-FAs from partially hydrogenated vegetable oils have well-established adverse effects and should be eliminated from the human diet (Michas et al., 2014). Palm oil may be an unhealthy fat, because of its high saturated FA content. Meta-analysis of 51 dietary intervention studies showed both favorable, and unfavorable changes in coronary heart disease and cardiovascular disease risk markers when palm oil was substituted for the primary dietary fats, whereas only favorable changes occurred when palm oil was substituted for *trans*-FAs (Fattore et al., 2014).

Higher degree of FA unsaturation is therefore a desirable characteristic to alter in palm oil. Iodine index, commonly used as unsaturation measure, varies from approximately 50–60% in *E. guineensis*, highest values measured for La Mé variety (Montoya et al., 2014), but was found to be anywhere between 70 and 80% in *E. oleifera* (Chavez and Sterling, 1991). Amongst the *E. oleifera* accessions, the unsaturated FA content ranges from 47 to 69% for C18:1, 2 to 19% for C18:2, and 0.1 to 1.2% for C18:3. Interspecies *E. oleifera* × *E. guineensis* hybrids planted in Latin America have a mid-parent phenotype with iodine index varying from 58 to 71% (Ong et al., 1981). Nineteen QTL’s controlling FA composition were identified in the interspecies pseudo-backcross populations (Montoya et al., 2014). Importantly for breeding purposes, work of Montoya et al. (2014) indicates that FA composition is not linked to biomass yield traits. Mapping intra-gene SNPs in candidate genes related to the oleic acid C18:1 biosynthesis, supported several QTL’s underlying acyl-ACP thioesterase type A (FATA) and Δ9 stearoyl-ACP desaturase (SAD) (Montoya et al., 2013). Genome sequence analysis identified the oil palm gene repertoire playing a role in FA biosynthesis, TAG assembly, carbon fluxes, fruit ripening and regulators of these processes (Singh et al., 2013b). In combination with the high-density genetic map (Ting et al., 2014) and further intra-gene SNP characterization, introgression of high oleic acid content from *E. oleifera* into varieties of *E. guineensis* is becoming a reality within a reach.

An alternative approach to increase oleic acid content at expense of palmitic acid relies on genetic engineering of key enzymes for palmitic acid synthesis, β-ketoacyl-ACP synthase II (KAS II) or palmitoyl-ACP thioesterase (Sambanthamurthi et al., 2009). KAS II activity was shown to be positively correlated with unsaturated FA content across palms from PORIM germplasm in Malaysia (Sambanthamurthi et al., 2009). Reducing *Arabidopsis* KAS II levels was found sufficient to convert its oilseed composition to that resembling palm-like tropical oil (Pidkowich et al., 2007). Mesocarp specific over-expression of KAS II gene and anti-sense RNA suppression of palmitoyl-ACP thioesterase have been undertaken and currently await evaluation (Sambanthamurthi et al., 2009).

The freshly pressed unrefined palm oil is also known as red palm oil due to its deep orangey-red color. Large amounts of carotenoids, predominantly α- and β-carotene, in a range 180–2500 μg g^-1^ mesocarp dry weight are measured in African oil palm populations (Rajanaidu et al., 2000; Tranbarger et al., 2011). Even higher contents up to 4000 μg g^-1^ mesocarp characterizes American oil palm accessions, whereas F1 interspecies hybrids have a mid-parent values of carotenoid contents (Sambanthamurthi et al., 2000). In terms of retinol equivalents (RE), standard batches of red palm oil have seventeen times more of β-carotene than carrots. A few grams, i.e., 1.5–6.5 table spoons, of red palm oil provides approximately 600 RE of β-carotene^17^, which is sufficient to meet daily vitamin A requirements in humans and to prevent childhood blindness from vitamin A deficiency (Burri, 2012). Indeed, worldwide interventions studies demonstrated usefulness of oil palm supplementation to improve vitamin A status; a deficiency commonly experienced by poor communities in Asia, Africa and South America (Rice and Burns, 2010). Red palm oil is also rich in tocotrienol, which is an unsaturated form of natural vitamin E. Tocotrienols have health benefits due to antioxidative, antihypercholesterolemic, and antiangiogenic effects on disease prevention (Wong and Radhakrishnan, 2012). *E. guineensis* population having high vitamin E content are available as well (Kushairi et al., 2004).

Oil content, composition and oil accumulating cells types are major traits of economic interest (Durrett et al., 2008). To decipher molecular mechanisms that underpin those traits, oil palm fruit development represents an excellent experimental model to apply “omics” trait dissection. Correlation analysis of the transcriptome and metabolome data has been performed on mesocarp samples harvested at multiple time-points during fruit development (Bourgis et al., 2011; Tranbarger et al., 2011). For comparative purposes and to advance understanding of the carbon partitioning between storage carbohydrates and TAG, similar data sets were generated in date palm (*Phoenix dactylifera*), a closely related palm species that accumulates almost exclusively sugars rather than oil in fruit mesocarp (Bourgis et al., 2011). Similar conclusions were drawn by both studies (Bourgis et al., 2011; Tranbarger et al., 2011). The transcript abundance of the FA biosynthetic machinery was remarkably coordinated with oil deposition in mesocarp tissues during fruit maturation. In contrast, TAG assembly pathway enzymes showed very low or the lack of up-regulation during fruit maturation, indicating that TAG assembly is not rate limiting for oil accumulation. The comparative co-expression analysis with transcriptomes of *Arabidopsis*, corn and date palm further implicated the oil palm APETALA2 (AP2)/ETHYLENE RESPONSE FACTOR family transcription factor EgWRI1-1 in regulation of FA accumulation. EgWRI1-1 is homologous to WRINKLED1, a transcriptional regulator of glycolysis and FA synthesis in *Arabidopsis* embryos (Cernac and Benning, 2004). Interspecies genetic complementation indicated that palm and *Arabidopsis* genes could be functional orthologs (Ma et al., 2013).

Oil content and composition differs between oil palm fruit mesocarp, endosperm and embryo. At 5 months after pollination, dry mass of endosperm contained 50% of oil in which lauric acid (C12:0) was predominant FA. The major FAs of mesocarp oil were palmitic acid (C16:0) and oleic (C18:1) acids. The oil palm embryo also stored up to 27% of oil, which contained 25% of linoleic acid (C18:2) (Dussert et al., 2013). To understand the mechanisms behind such differences in oil content and FA composition, transcriptome and lipid profiles were compared during development of oil palm fruit. Accumulation of lauric acid in endosperm relied on up-regulation of a acyl–acyl carrier protein thioesterase and TAG assembly enzymes isoforms (Dussert et al., 2013). Three paralogs of WRINKLED1 were proposed as candidate regulators determining different lipid profiles. In agreement with (Tranbarger et al., 2011), *EgWRI1-1* was found to operate in mesocarp. *EgWRI1-2* and *EgWRI1-3* were predominantly expressed in endosperm. Interestingly, embryo did not express either of *EgWRI1* paralogs (Dussert et al., 2013).

To provide new breeding material, targeted approaches, such as Ecotilling (Till et al., 2006) can be applied to screen oil palm germplasm collections for the loss or gain of function alleles in the identified subsets of lipid biosynthetic and regulatory genes. There are very few reports on induced mutagenesis in oil palm (Corley and Tinker, 2003). We are not aware of any chemically mutagenized oil palm populations that can enable standard TILLING (Till et al., 2006). Due to the 4-years-long seed-to-seed cycle, the practicality of such oil palm population of a few thousands trees that will live for the next 100 years appears to be doubtful. This view may change, pending the progress with production of doubled haploids from oil palm microspores (Indrianto et al., 2014). Mutagenizing microspores may result in a “tilling” population of trees homozygous at all genetic loci the analysis of which will add supportive evidences for the “omics” data.

## Oil Palm Tree Architecture

Harvesting oil palm is expensive in manual labor, difficult task, compared with the ease of combine-harvesting arable crops (Corley and Tinker, 2003). The radical changes to the oil palm tree architecture are needed to enable development of harvesting machines. The major utility in harvesting mechanization were found to be palm height and bunch stalk length and thickness (Le Guen et al., 1990). A number of problems arise as palms age on a plantation. Fruit harvest is complicated when oil palm trees are taller than two-three meters. Cutting bunch stalk becomes physically challenging. Bunch fall bruises fruits many of which detach, demanding additional labor effort to collect lose fruits from the ground. Bruising activates TAG hydrolysis that lower oil quality. It is more difficult to assess the bunch ripeness. Though oil palm is a long lived species, replanting is thought to be required for plantations 20–25 years of age (Corley and Tinker, 2003).

In relation to the environment and genetic makeup, African oil palm accessions have height increment of 45–75 cm a year. Annual height increment of *E. oleifera* may be only 5–10 cm. Interspecific F1 hybrids between *E. guineensis* and *E. oleifera* have a mid-parent growth phenotype of 15–25 cm annual height increment (Corley and Tinker, 2003), indicating a useful gene introgression source.

Intraspecific variation enabled Malaysian breeding programs to develop PORIM series of dwarfish palms with a yield potential of 7t and annual height increment of 40 cm (Rajanaidu, 1994; Rajanaidu et al., 2000). Hybrids from crosses of Bamenda and Ekona *E. guineensis* accessions developed by ASD Costa Rica^18^ are slower growing (45–50 cm/year) and are also known for overall high cold and drought tolerance.

To find QTLs that control the tree growth rates, two breeding populations of oil palm were used for linkage mapping (Lee et al., 2015). For selected genotypes of the *dura* and *pisifera* parents, the heights of the 6-year-old *tenera* palms in F1 populations were distributed from 71.0 to 180 cm with an average of 137.6 cm. The QTL positioned on a linkage group 5 explained 51.0% of the phenotypic variation, suggesting that it should play a major role in height variation of selected palm genotypes. Oil palm genome sequence indicated that QTL is located more precisely within 65.6 kb region that includes eight genes, of which the gene encoding asparagine synthase-related protein is thought to be responsible for the tree height variation amongst analyzed *teneras* (Lee et al., 2015). Along with glutamine synthases, asparagine synthases have an important role in nitrogen assimilation and allocation within the plant. Interestingly, the ectopically expressed pine glutamine synthase accelerates the growth of the poplar trees (Man et al., 2011).

In *E. oleifera* × *E. guineensis* F1 hybrid population, a wild palm was discovered that in addition to short trunk, had relatively short leaves due to spontaneous heritable change in leaf length. Derived breeding program resulted in commercial COMPACT^19^ varieties sold as clones. Compared to 7–8 meters long leaves of standard *tenera* hybrids, COMPACT palm leaves are 6.5 meters long, which allows a very high density planting, 180–200 trees per hectare. COMPACT palms have < 40 cm/year height increment. Micropropagated clones are rather expensive. Fortunately, the leaf length trait appears to be semi-dominant. Hybrids between COMPACT palms and standard *E. guineensis* lines, such as Deli, Ghana and Nigeria, have 6.6–6.9 meters long leaves and can be planted at density of 170 trees per hectare, which is still higher than industry standard of 138–143 palms/hectare (Corley and Tinker, 2003). As compared to clones, seeds of such hybrids are more affordable for smallholder farmers, who can find such genetic material as an opportunity to increase production and make better use of scarce land resources (Alvarado et al., 2010).

The increases in wheat and rice yields during the “Green Revolution,” were enabled by the introgression of dwarfing traits into the plants (Hedden, 2003). The “Green Revolution” genes showed the central role of gibberellin (GA) in the control of plant stature. Wheat *Reduced height (Rht)* genes interfere with the GA signal transduction pathway. The rice *semidwarf1* (*sd1*) gene impair the GA biosynthesis. *Arabidopsis GA5* gene is the ortholog of rice “Green Revolution” gene *SD1* (Barboza et al., 2013). Importantly, semidwarf individuals found in natural *Arabidopsis thaliana* populations were 21 different independent loss-of-function mutations at *GA5*. Semidwarfness had no obvious general tradeoff affecting *Arabidopsis* plant performance traits (Barboza et al., 2013). Semidwarfism transgenes modifying GA, promoting root growth and enhancing morphological diversity, have been tested in hybrid poplar trees (Elias et al., 2012). Analysis of mutants in cereals further implicated brassinosteroids in the control of plant architecture (Dockter and Hansson, 2015). These findings have direct implications to the gene-centered analysis of oil palm natural variation and tree architecture engineering.

## Preventing (Post)Harvest Losses

The chemical properties of oils used in commerce are extremely important. The hydrolysis of TAG and release of free FAs has a strong impact on the quality of commodity oil, because free FA content above five percent is thought to be unfit for human consumption (Ebongue et al., 2008). Oil palm mesocarp contains a highly active lipase that within five minutes can bring the free FA content to 30% in crushed tissue. The biological function of lipase in palm fruit mesocarp is uncertain. Importantly, TAG hydrolysis does not occur in undamaged fruits. It is critical to reduce fruit bruising before they reach the oil mill where the first step of post-processing is high pressure steam sterilization to inactivate both palm, and microbial lipases (Corley and Tinker, 2003).

To gain flexibility for post-harvest fruit processing and extended ripening for increased yields, elite oil palm lines with a low lipase (LL) were selected. Oil pressed from LL fruits had substantially less free FAs than standard genotypes (Ebongue et al., 2008). *E. guineensis LIPASE1*, *EgLIP1* gene associated with the LL trait has been identified, allowing marker-based introgression of the LL trait into any elite oil palm genotypes (Morcillo et al., 2013). Approximately 30% of oil palm cultivation world-wide, and up to 80% in Africa, is in smallholder farms. To extract the best quality oil, farmers have only limited period of time to deliver their produce to the oil extraction mills. Commercialization of the LL trees is estimated to generate economic gain of almost billion US dollars per year (Morcillo et al., 2013).

*Elaeis oleifera* has naturally very LL activity in fruit mesocarp, whilst interspecific *E. oleifera* × *E. guineensis* hybrids are promising crosses with less lipase activity (Cadena et al., 2013). Overall it appears that several *E. oleifera* traits, e.g., *virescens* fruits, LL, lack of methyl chavicol biosynthesis that could have been interpreted as loss-of-function mutations, behave at least as co-dominant or dominant alleles in interspecies hybrids. Molecular identification of genes controlling such traits (Morcillo et al., 2013; Singh et al., 2014) will help to reach mechanistic understanding of interspecies genome interactions.

Shedding of ripe fruits from the bunches before they reach the factories is an important source of harvest losses (Osborne et al., 1992). The development of the fruit abscission zone takes place at the base of subtending fruit. It is a two-stage process involving primary and adjacent abscission zones. Abscission zones contain very low amount of methylated pectin and high levels of polygalacturonase (PG) activity that is involved in the depolymerisation of the cell wall pectin homogalacturonan (Henderson et al., 2001). The oil palm PG family comprise at least 14 genes, of which ethylene-inducible *EgPG4* is the most highly expressed in the fruit base (Roongsattham et al., 2012). Altogether, we may anticipate breeding of palms with delayed, if not abolished, fruit shedding. The question becomes how to assess the fruit bunch ripeness for such palms in practice?

The majority of commercial *teneras* have *nigrescens*, i.e., anthocyanin colored, fruit exocarp. To determine that bunches on *nigrescens* palms are ripe, harvesters rely on the presence of detached fruits on the ground (Corley and Tinker, 2003; Singh et al., 2014). It is thought that scoring the profound change in color from green to bright orange upon ripening in *virescens* fruits could be an alternative solution to assess bunch ripeness in the field (Sambanthamurthi et al., 2009). The *E. guineensis VIRESCENS* (*VIR*) gene is a R2R3-MYB transcription factor (Singh et al., 2014). The dominant-negative *virescens* phenotype is explained by the expression of the VIR protein isoforms truncated at carboxyl-terminus, which is likely to function as transcription activation domain. Interestingly, *E. oleifera* naturally has *virescens* fruit phenotype. Singh et al. (2014) cannot identify *VIR* homolog in a current *E. oleifera* draft genome assembly, indicating that *virescens* phenotype of *E. oleifera* fruit could be explained by a natural deletion mutation.

## Breeding for Expanded Cultivation Range

Standard cultivation range of oil palm commercial varieties lays within 20° of the equator (Corley and Tinker, 2003). The cultivation range, as well as the breeding challenges, are expected to evolve due to the climate change (Paterson et al., 2013). Expansion of cultivation range to sub-tropical regions (Lei et al., 2014) could lower the negative impact of the crop on tropical biodiversity.

Cold tolerance was observed in natural oil palm groves situated at 1000–2000 meters above sea level in the Bamenda Highlands of Cameroon and in Kigoma District, Tanzania (Blaak and Stirling, 1996). A breeding program by ASD de Costa Rica^20^ and FAO^21^, using Bamenda and Kigoma germplasms, led to new cold tolerant commercial varieties that in addition showed precocity when planted at sea level, producing fruit at 2 years after planting (Chapman et al., 2003). With a goal to gain molecular understanding of cold stress response and to expand African oil palm cultivation to sub-tropical regions, including Hainan province located in the southern China, the cold stress response in oil palm was analyzed by deep RNA sequencing (Lei et al., 2014). Work revealed 51,452 expressed sequences from *E. guineensis*. Transcriptome data analysis resulted in discovery of 5791 gene-based simple sequence repeats (SSRs) markers of which 916 distinguished genes differentially expressed in response to cold stress (Xiao et al., 2014).

Breeding African oil palm for stress tolerance, especially drought tolerance, has been found to be challenging (Corley and Tinker, 2003). The alternative to lower the environmental footprint of the crop, whilst meeting the growing demand for vegetable oils, is to domesticate other oleaginous palm species that have different profile of ecophysiological adaptations.

To address the problem of fatal yellowing disease that restricts oil palm cultivation in Latin America, breeders of the ASD Costa Rica introduced a new hybrid variety AMAZON. The mother trees are *E. oleifera*, originating from wild palms indigenous to the Manaus region (Amazonas state, Brazil). The *pisifera* parents were selected from the progeny of *E. oleifera* × *E. guineensis* interspecies hybrid backcrossed to *E. guineensis*. In the AMAZON hybrid, *E. guineensis* genome is smaller than haploid in size. This work is a pioneering step toward domestication of *E. oleifera* through gene introgression for higher yield.

*Acrocomia aculeata* known as macaw palm, or macaúba in Portuguese, is particularly interesting for the development of a new oleaginous crop both because of high productivity potential, simpler harvest, and ability to grow in arid sub-tropical areas (Pires et al., 2013). Whilst collecting *E. oleifera* accessions in Latin America, Malaysian breeders added to their *ex situ* germplasm collection several other oleaginous palms, such as *Oenocarpus* spp., *Bactris gasipaes* (Corley and Tinker, 2003). It is beyond the doubt that the recent advances in understanding the oil palm genome, physiology, identification of key genes controlling productivity and TAG biosynthesis will accelerate the domestication breeding programs.

## Concluding Remarks

In this review we focused on the oil palm genetic diversity and how modern genomics tools could contribute both to the basic understanding of the physiology, metabolism and development of this remarkable crop, and to accelerate breeding of the high yielding varieties with a tailored oil composition.

Many of the discussed traits could be engineered using genetic modification of the crop (Murphy, 2014). Of particular interest is the success with regeneration of plants from protoplast cultures that were shown to be the superior starting material for PEG-mediated DNA transfection and microinjection (Masani et al., 2014). This system could be an excellent recipient to implement genome editing technology (Joung and Sander, 2013; Shan et al., 2013). For instance, instead of time consuming gene introgression, *EgLIP1* gene can be destroyed in high oil yield *tenera* elite palm, or its *dura* and *pisifera* parents. High oleic acid content and the ability to synthesize polyunsaturated fatty acids (PUFA) could be similarly engineered using simpler *Agrobacterium*-mediated genetic modification (Murphy, 2014). Those traits in combination with high pro-vitamin A and vitamin E content could result in new palm varieties for the extraction of the virgin red palm oil of unprecedented nutritional quality serving millions, if not billions of people from impoverished countries. As of today, palm oil is the only non-GMO oil on a global market, which some activists believe increases the palm oil value.

Plant breeders will continue with the efforts to increase primary crop productivity, however, the immediate challenges in closing the yield gap lay in providing smallholder farmers with the access to the best planting material, with balanced application of fertilizers and overall corrects agronomical practices. The negative impact of the crop on biodiversity is undeniable; the high yielding varieties are available to spare the land. It is the socio-economical drivers, governmental decision and environmental activists views that make oil palms the “Palms of Controversies” (Rival and Levang, 2014).

### Conflict of Interest Statement

The authors declare that the research was conducted in the absence of any commercial or financial relationships that could be construed as a potential conflict of interest.

## Footnotes

1 http://www.wri.org

2 http://www.greenpeace.org

3 http://www.rspo.org

4 http://faostat3.fao.org/home/E

5 http://www.indexmundi.com

6 http://iopri.org/

7 http://www.cirad.fr/

8 http://www.mpob.gov.my/

9 http://www.fao.org/docrep/013/i1500e/i1500e00.htm

10 https://www.embrapa.br/en/amazonia-ocidental

11 http://www.palmelit.com/en/

12 https://www.embrapa.br/en/amazonia-ocidental

13 http://genomsawit.mpob.gov.my/genomsawit/

14 http://www.palmelit.com/

15 http://www.asd-cr.com/

16 http://www.asd-cr.com/

17 https://hungermath.wordpress.com/2013/01/02/red-palm-oil-as-a-source-of-beta-carotene/

18 http://www.asd-cr.com/

19 http://www.asd-cr.com/

20 http://www.asd-cr.com

21 http://www.fao.org
